# Supplementary figures and images for: Immunization of turkeys with Clostridium septicum alpha toxin-based recombinant subunit proteins can confer protection against experimental Clostridial dermatitis
Source: PLoS One. 2024 Apr 29;19(4):e0302555. doi: 10.1371/journal.pone.0302555 (PMC11057757; doi:10.1371/journal.pone.0302555)

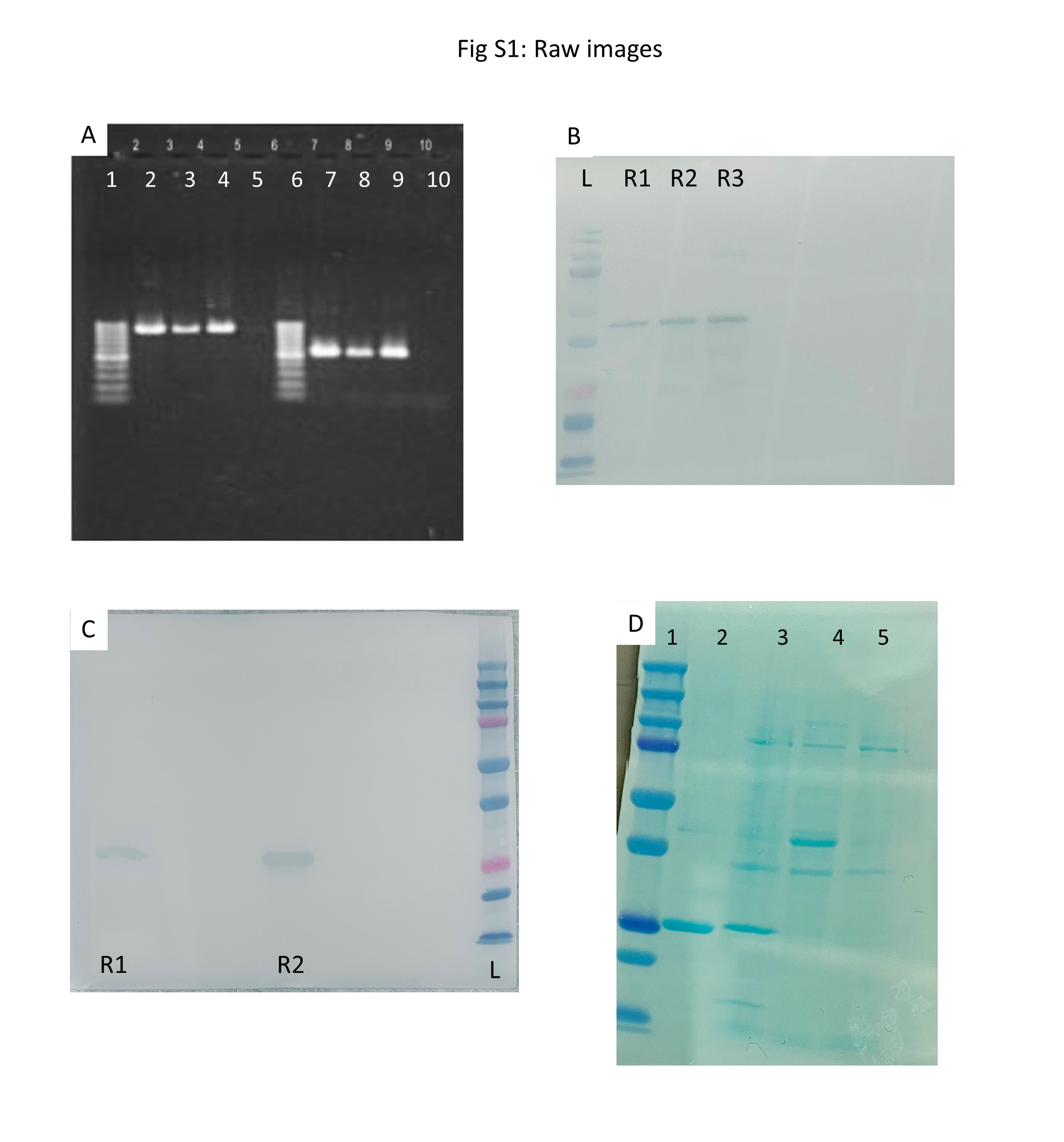

Supplement: S1 Fig — Pic A: Lanes 4 (912bp), 5 (neg ctrl), 6 (ladder) and 7 (531bp) were cropped from the gel pic below for the main Fig 1B. Pic B: Lanes R1, R2 and R3 are the purified ntATX-D1. To make the main Fig 1C, lane R3 was cropped and used along with the protein ladder L. Pic C: Lanes R1 and R2 are the purified ntATX-D2. To make the main Fig 1C, lane R2 was cropped and used along with the protein ladder L. Pic D: Lanes 2 and 3- ntATX-D2, Lanes 4 and 5- ntATX-D1, Lane 1- Ladder. To make the main Fig 1D, the lanes 1, 2 and 4 were cropped. (TIF) [file pone.0302555.s001.tif]

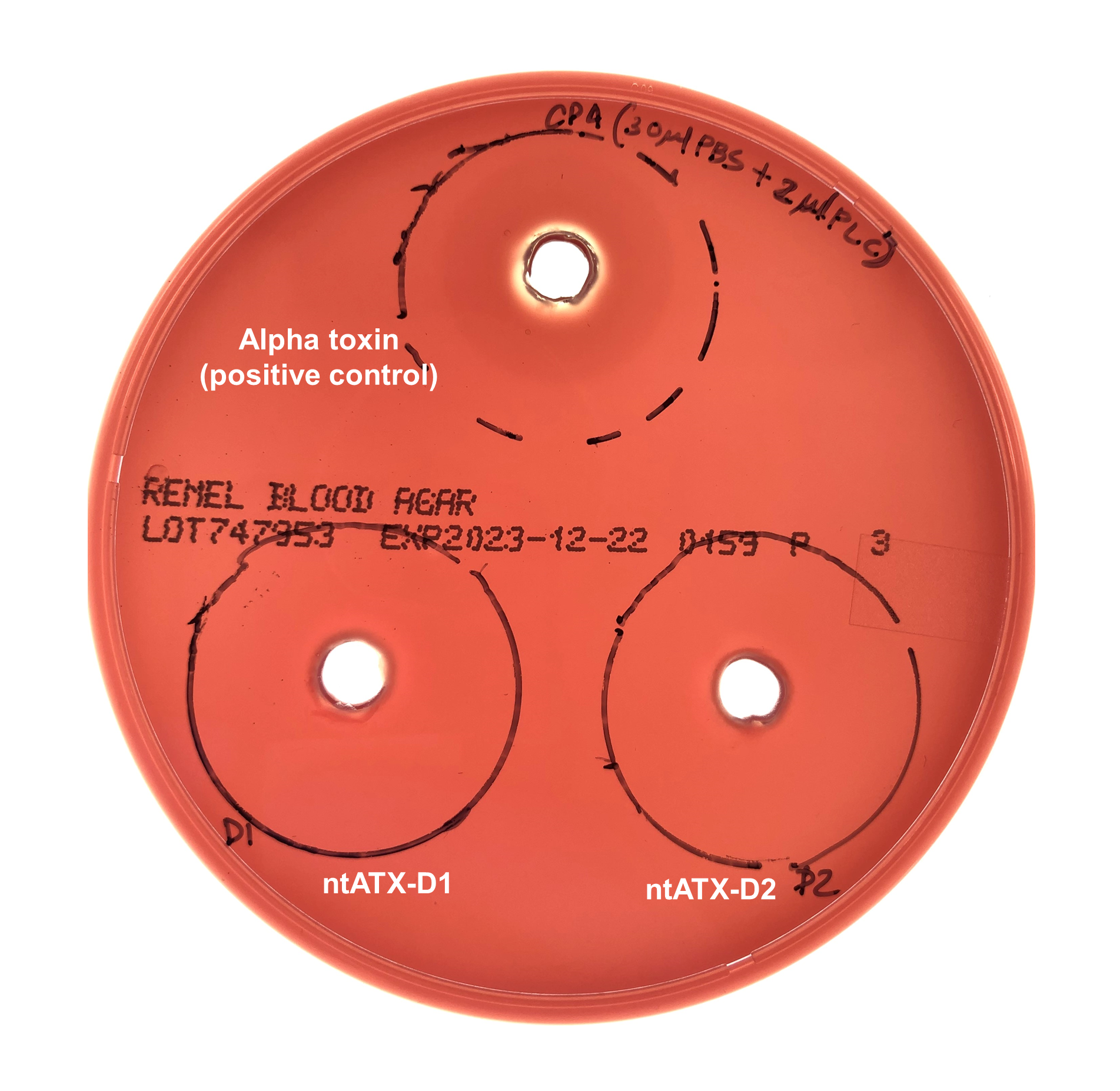

Supplement: S2 Fig — The hemolytic activity of purified ntATX-D1 and ntATX-D2 was evaluated using 5% defibrinated sheep blood agar plates. Wells were punched into the agar and loaded with 30 μL of purified proteins (10 μg/well) and incubated at 37°C overnight. The absence of a zone of hemolysis indicated the non-hemolytic activity of the proteins. As a positive control for hemolysis, the purified C. perfringens alpha-toxin was used. (TIF) [file pone.0302555.s002.tif]

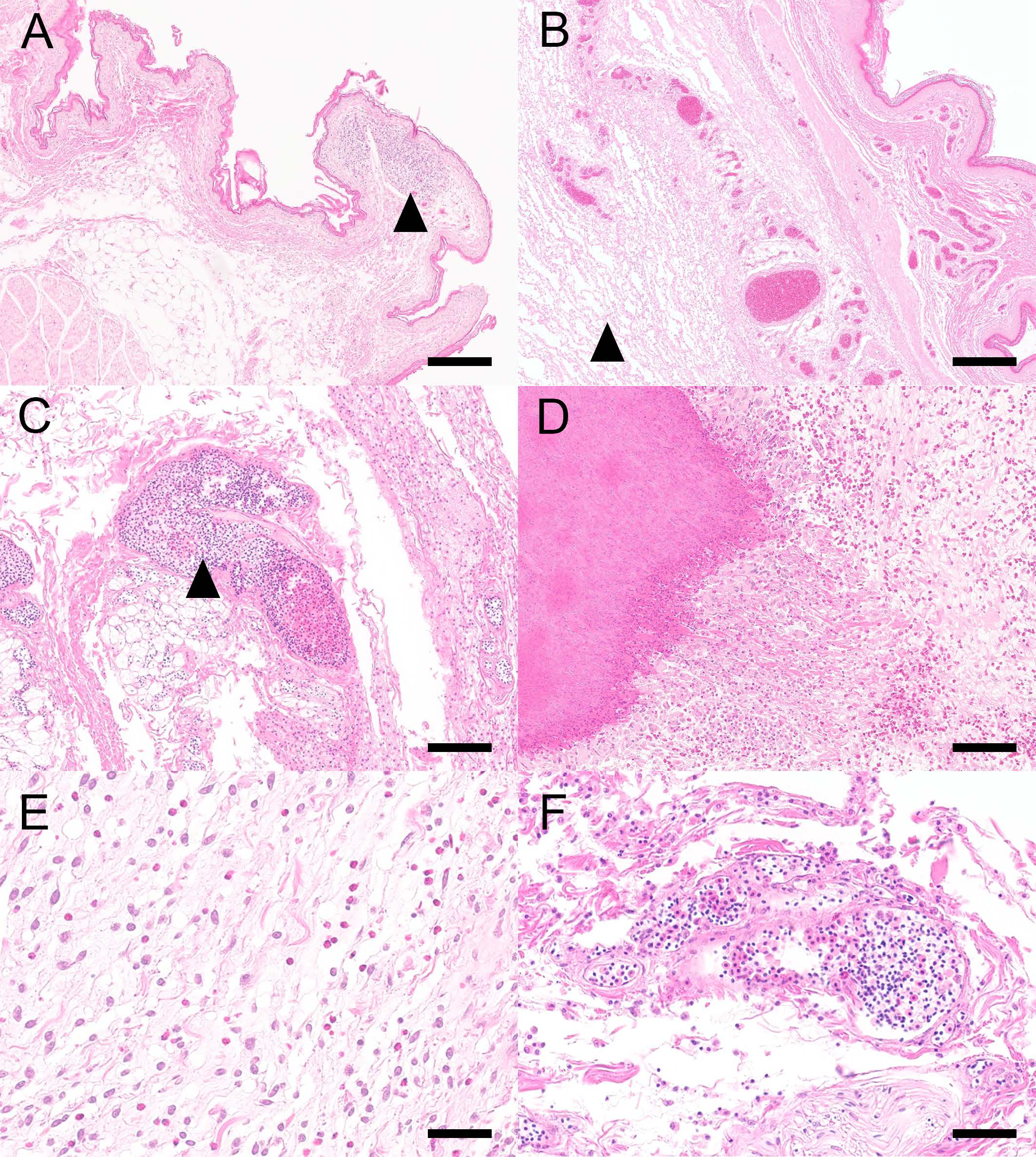

Supplement: S3 Fig — (A) Negative control. Occasional samples had focal, mild, often perivascular lymphocytic and heterophilic inflammation in the superficial dermis (arrowhead), affecting less than 5% of the tissue (lesion scores = 2). Bar = 150 μm. (B) Positive control. The dermis is markedly expanded by fibrin and edema (arrowhead), consistent with a lesion score of 4. Bar = 325 μm. (C) ntATX-D1 vaccinated bird. Skin shows cell lysis of erythrocytes (arrowhead) multifocally affecting 30–75% of the tissue, consistent with a lesion score of 4. Bar = 100 μm. (D) ntATX-D1 vaccinated bird. The deep dermis contains a discrete caseous granuloma characterized by an outer rim of macrophages and heterophils surrounding an inner, hypereosinophilic core of caseous necrosis. Bar = 100 μm. (E) ntATX-D2 vaccinated bird. The deep dermal collagen is separated by abundant fibrin and edema, with small numbers of scattered heterophils. Bar = 50 μm. (F) ntATX-D2 vaccinated bird. Severe cell lysis of erythrocytes within blood vessels, with marked expansion of the adjacent deep dermis by abundant fibrin and edema. Bar = 50 μm. (TIF) [file pone.0302555.s003.tif]

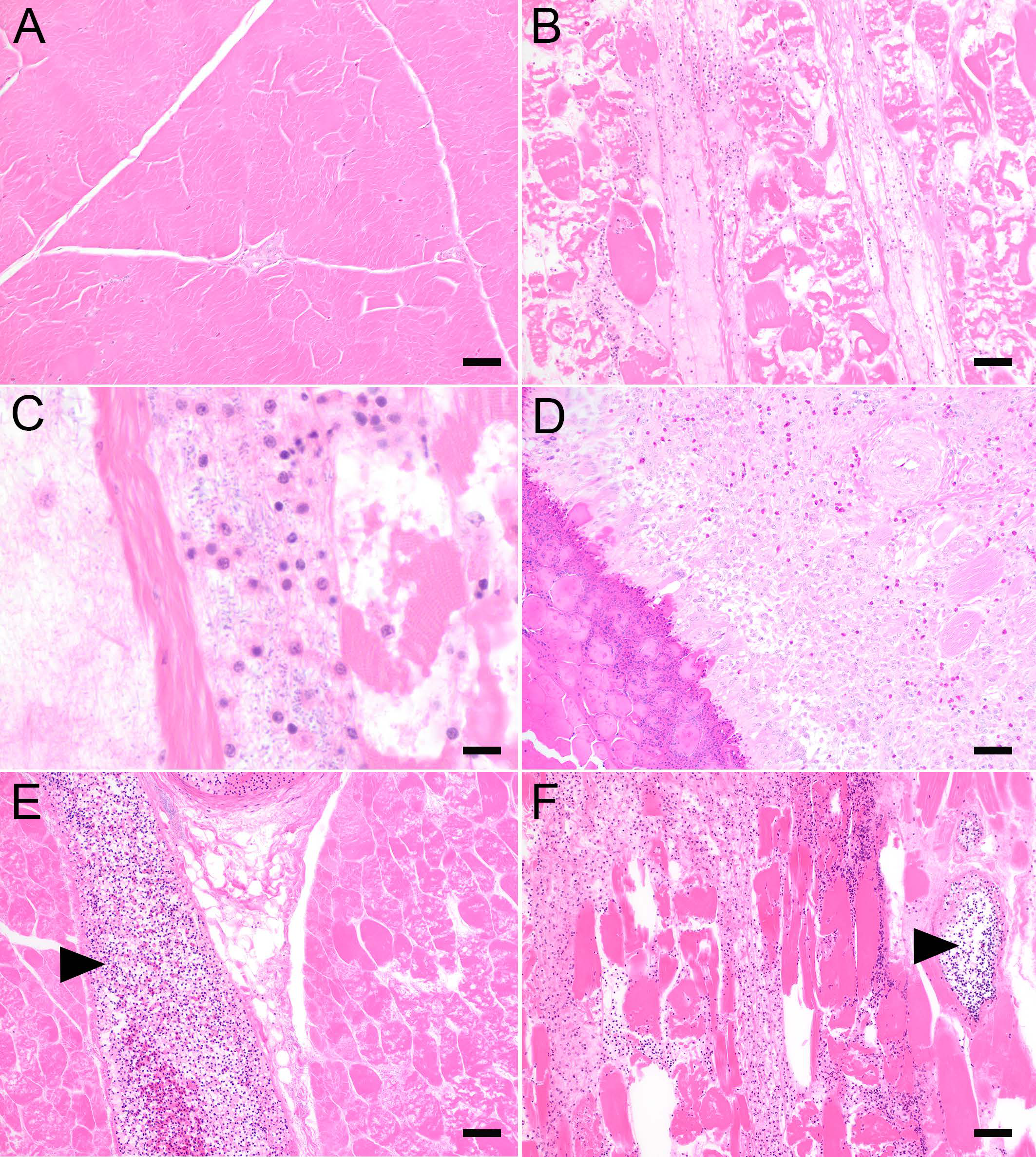

Supplement: S4 Fig — (A) Negative control. No lesions were present within any of the negative control birds, with scores equivalent to 1 for all categories. Bar = 50 μm. (B) Positive control. Skeletal myofibers often exhibit monophasic sarcoplasmic fragmentation and hypereosinophilia with loss of cross-striations, affecting over 75% of the myofibers (lesion score = 5). Myofibers are often separated by fibrin and edema (lesion score = 4) admixed with scattered cellular lysis, affecting 30–75% of the tissue. An inflammatory infiltrate is absent. Bar = 50 μm. (C) Positive control. Foci of cell lysis, fibrin, and edema frequently contain abundant large bacterial rods. Lesion score was 4 for bacteria, with bacteria scattered extensively throughout 30–75% of the section. Bar = 10 μm. (D) ntATX-D1 vaccinated bird. Skeletal myofibers are replaced by a focally extensive caseous granuloma, characterized by an outer rim of abundant macrophages, multinucleated giant cells, and fewer heterophils and lymphocytes (granuloma lesion score = 4). The rim surrounds an inner core of caseous necrosis of skeletal muscle, with abundant cellular debris and brightly eosinophilic, necrotic myofibers (caseous necrosis lesion score = 4). Bar = 50 μm. (E) ntATX-D1 vaccinated bird. Erythrocytes within and surrounding vessels exhibit cell lysis multifocally in up to 25% of the section, consistent with a cell lysis lesion score of 3 (arrowhead). Myofibers exhibit similar degeneration and necrosis as in the positive controls. Fibrin and edema multifocally separate myofibers. Bar = 50 μm. (F) ntATX-D2 vaccinated bird. Similar cell lysis, fibrin, edema, and myopathy are visualized as described in the positive control and D1 vaccinated bird. Bar = 50 μm. (TIF) [file pone.0302555.s004.tif]

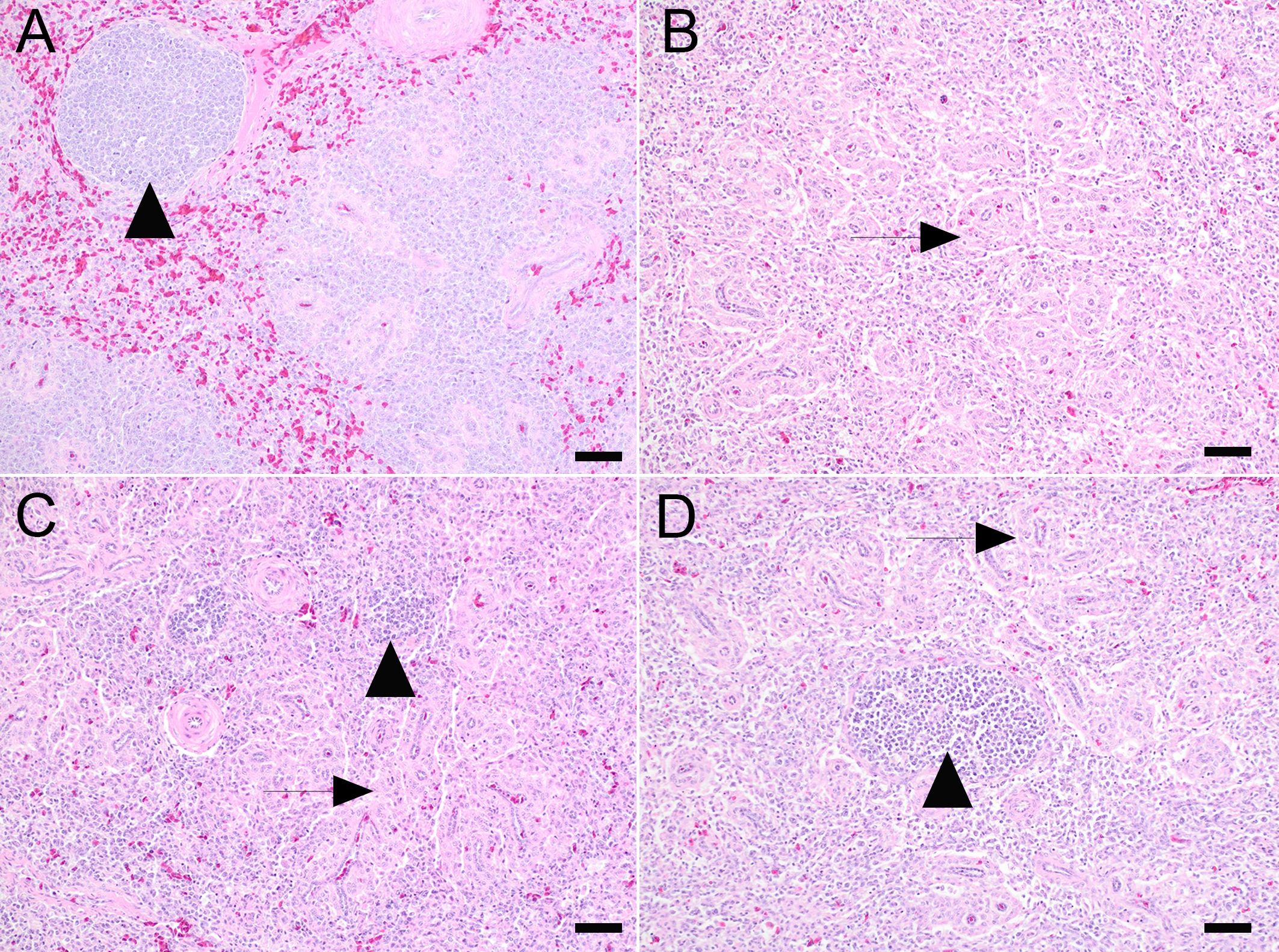

Supplement: S5 Fig — (A) Negative control. No lesions are identified with the negative control group. The arrowhead represents a bursa dependent lymphoid nodule. (B) Positive control. A subset of birds had unapparent bursal dependent nodules, depletion of lymphocytes around sheathed venules, and reticular hyperplasia. Scores in this animal for lymphoid depletion and reticular hyperplasia (arrow) were a 4 with 30–75% of the tissue involved by multifocal lesions. (C) ntATX-D1 vaccinated bird. Occasional birds had atrophy of bursal dependent lymphoid nodules (arrowhead) and of lymphocytes around sheathed venules (lymphoid depletion lesion score = 3). Reticular hyperplasia was equivalent of a lesion score 4, affecting 30–75% of the tissue. (D) ntATX-D2 vaccinated bird. Occasional birds had lymphoid depletion (arrowhead) and reticular hyperplasia (arrow). Bars = 50 μm. (TIF) [file pone.0302555.s005.tif]
