# Supplementary material for: Immunization of turkeys with Clostridium septicum alpha toxin-based recombinant subunit proteins can confer protection against experimental Clostridial dermatitis
Source: PLoS One. 2024 Apr 29;19(4):e0302555. doi: 10.1371/journal.pone.0302555 (PMC11057757; doi:10.1371/journal.pone.0302555)
Supplement: Attachment — Submitted filename: Response to Reviewer-1 comments.docx [file pone.0302555.s006.docx]

**Reviewer #1 Comments to the Author**

Overall, the manuscript is well written, and the data is quite interesting. However, there seems to be lack of clarity over the induction of the different arms of the immune system by subunit vaccines. The way the authors approached the explanation of the results and conclusion, it’s not clear which (innate, cell or humoral) immunity has been induced. They can argue that all of them were induced but testing for cytokines in vaccinated birds at termination (of experiment??) doesn’t sound rational. I would suggest that the authors clearly distinguish which arms of the immune system has been induced.

**Response**: *We thank the reviewer for this very insightful comment and certainly, agree that there has been some lack of clarity in our discussion of findings related to the protection mechanisms. Although it’s difficult to affirmatively state, based on our findings, as to which arm of the immune system may have played a vital role in protection against CD, we do believe that it’s likely the adaptive arm (antibody and T cell responses) that plays a key role in subunit immunization-induced protective immunity.*

*Since we couldn’t evaluate the macrophages and γδT cells in this study (as we always have done in our chicken Necrotic Enteritis works) due to the unavailability of turkey-specific antibodies for immunophenotyping, we’re somewhat unsure about inferring on the innate arm of the immunity. We looked at the cytokines primarily as a measure of inflammation and immunoregulation during infection and protection.*

*Considering the critical value attached to this reviewer’s comment, we have now revised the parts of the discussion as well as our conclusion points.*

**L56 – 57**: Are you referring to a commercial availability here? Please be specific because research has been ongoing for a while to develop vaccine with some vaccines showing promise. Also, please specify here if you are only referring to vaccines for poultry.

**Response**: *Yes; Thank you and suggested correction has now been made in the revised manuscript.*

**L158**: Please provide more information about the strain here. You have cited “5” but more information about how “hot” the strain is would be required. What is the expected mortality/morbidity when you challenge the birds with this strain. Please add this information.

**Response**: *Thank you. While this manuscript was being prepared, we had another manuscript under revision with Veterinary Immunology and Immunopathology journal, which addressed the characterization of immune responses against C. septicum strains, including Str. B1, and was very recently accepted and available online. In the revised manuscript, we have now provided additional information, as per the reviewer’s comment, along with citing the published manuscript (added as ref# 10).*

**L192**: At which time point were the tissues collected? I can only assume that you did qPCR on tissues collected at termination (but what does termination mean?). Is it when the birds died, or you had a specific age of birds? Please make this clear.

**Response**: *We thank the reviewer for this comment and realize the unclarity of information provided. We have now revised the materials and methods section and included more clear information incorporated along with the experimental challenge and necropsy information.*

**L242**: For the ELISA section, what was the starting concertation of serum? Was it whole serum or you diluted it to a certain point first? It may be shown in your plots, please specify here. Also, I would ask the authors to consider using the following paper to do a statistical analysis of their ELISA data. You can also do End-Point titre method.

**Response**: *Thank you for this important comment. The starting dilution was 1:200 and we have added this information in the suggested methods section. Although we did not see the link to the paper suggested by the reviewer (may have forgotten to add the link), we did get the reviewer’s point of view. Traditionally, in the past, we have used the end-point titer method for such investigation related to immunogenicity of the vaccine antigen. However, we didn’t have a good positive control serum this time to run to ensure its validation, which is a critical need. We tried using the infected serum collected for our previous studies (PMID:* [*37140115*](https://pubmed.ncbi.nlm.nih.gov/37140115/) *and* [*38340537*](https://pubmed.ncbi.nlm.nih.gov/38340537/)*) but wasn’t satisfactory. Therefore, to report the seroconversion to vaccine antigens, we chose to plot the OD values, which were evident to suggest antigen-specific IgY response.*

*As per the reviewer’s suggestion, we have now included the statistical information in the methods, as well as uploaded the revised figure 9 depicting statistical significance.*

**L257**: Add the statistical analysis method you used for ELISA. Also, why didn’t you analyze your mortality? Can you do a survival analysis using Kaplan-Meier curves or hazard ratios? This is very important for the mortality data, which seems to be quite significant for your study.

**Response**: *As per the suggestion, we have added the statistical method, and also prepared a ‘survival chart’ to replace the previous mortality bar graph in Figure 2. We thank you for this comment and suggestion.*

**L270** – 282: This reads like a Materials and Methods. You have described these already. So, focus on the outcomes.

**Response**: *The text portion related to the comment has now been deleted*.

**L283** – L295: Same as Above

**Response**: *This portion relates to the Figure 1 ‘legend’ and we think it would be necessary to leave it as is to support the journal’s requirement for writing figure legends such that the figures can stand-alone without the necessity for the readers to search for the information in the methods section.*

**L297** – 302: Same as above. Why do you need to repeat this here?

**Response**: *The text portion related to the comment has now been deleted*.

**L355**: Here you are being selective on which treatment comparisons you’re reporting. For instance, for IL-1b, you compare the two controls, but for IL-6, you only compare NCx and D2-immunized birds. If you see IL-6, PCx and the immunized groups don’t seem to show significant differences. When you look at the figure for NCx and PCx, the bars are quite different, and you statistic should show significance. This is relevant because your immunization may not be the cause for higher IL-6.
Suggestion: NCx vs PCx comparison only shows you if you challenge worked. The comparing immunization to PCx should be reported. Here letter-based identification for statistical significance may be useful. Please to make the best out of your data.

**Response**: *We immensely thank the reviewer for this comment and apologies for the unclarity of information provided related to our statistical analysis. The following are the points of our response to this comment.*

1) *The statistical comparison related to gene expression, cellular response and histopathology data were not selective and the tests/analysis used multiple comparisons between all the 4 groups. We have now included more details in the methods section. For gross lesions, we purposefully used comparisons to only PCx considering the primary reason for protection assessment and that none of NCx birds had any lesions (as expected).*
2) *Some gene expression data that look significant were not found ‘statistically’ significant and we have re-checked these data. This perhaps is because we chose to use ‘multiple comparison’ stat tests, while comparing just two groups may yield different outcomes, which wasn’t considered in the present manuscript.*
3) *Considering reviewer’s excellent suggestion to have a letter-based identification of stat significance, we have now revised five Figures (# 4 through 8) in the revised submission of this manuscript. We also noticed two errors in the Median range bars in Figure# 3, we have it also revised for including in this revised submission.*

*We sincerely hope that our response to this comment is satisfactory to the reviewer.*

**L460**: Figure 8F not shown on figure

**Response**: *Thank you for this observation; we have rectified the error reflecting panel 8F, and also the 8G (for the graph related to MHC-II+ cells.*

**L480 – 484**: Please revise after you consider the statistical analysis

**Response**: *Thank you, again. We have now revised to reflect the suggestion made by the reviewer.*

**L535 – 538**: Good point. But you didn’t show this in the figure, not did you describe it in your “results” section

**Response**: *We didn’t quite get this comment since we have shown this in our figures. Namely, the gene expression of IL-1β in all tissues (skin in Figure 4, muscle Figure 5, CT Figure 6) except spleen, the IL-6 in muscle, IFNγ in CT was significantly upregulated in PCx (challenged) birds when compared to unchallenged NCx birds. These were described in the results section (lines 359-360, 376-377 and 392-393) of the original submission. However, in the discussion (line 537 of original submission), we found an error and removed ‘spleen’; hence, we thank the reviewer for asking us to confirm the statement.*

*We also believe that in case the reviewer has missed out our mention of these findings in the results section, it is most likely due to the figure legends inserted in the text soon after the figure results are described. This insertion was as per the journal’s manuscript submission instructions to authors. Therefore, we have now added the lines in the results text to demarcate the legends from the results description in the revised ‘track-changed’ version of the manuscript, while these lines are deleted in the ‘clean’ (no track changes) version of the manuscript. Bothe the versions are uploaded as per the journal instructions.*

**L550 – 553**: Compared to which treatment? Be specific

**Response**: *We had it mentioned ‘when compared to PCx group’ and perhaps, wasn’t noticed since it’s in the beginning of the sentence.*

**L559 – 562**: This conclusion may be a bit of a stretch. I can argue that because you’re analyzing innate immune response genes at “termination” (if my assumption is correct, this would be post-vaccination), then your lower innate genes could be manily because the birds are mounting strong antibody mendiated responses, hence not require innate mediated response. Given the fact that you’re using a subunit vaccine, this is highly likely. Please revise

**Response**: *Thank you. Some parts of the conclusion has been revised, as suggested.*

**L594 – 600**: Mortality is one of your strongest data. Why did you omit it from your overall conclusion?

**Response**: *Thank you for this comment. We have now revised the first sentence of the concluding points to reflect reviewer’s comment and suggestion.*
